# Cerebral infarction caused by a heart-breaking needle: a case report

**DOI:** 10.1186/s13256-015-0797-z

**Published:** 2016-02-03

**Authors:** Shaodong Ye, Lin Li, Qiuting Dong, Guogan Wang

**Affiliations:** 1 Center for Coronary Heart Disease, FuWai Hospital and Cardiovascular Institute, Peking Union Medical College and Chinese Academy of Medical Sciences, 167 Beilishi Road, XiCheng District, Beijing, 100037 P. R. China; 2 Center for Emergency and Intensive Medicine, FuWai Hospital and Cardiovascular Institute, Peking Union Medical College and Chinese Academy of Medical Sciences, 167 Beilishi Road, XiCheng District, Beijing, 100037 P. R. China; 3 Department of Cardiology, FuWai Hospital and Cardiovascular Institute, Peking Union Medical College and Chinese Academy of Medical Sciences, 167 Beilishi Road, XiCheng District, Beijing, 100037 P. R. China

**Keywords:** Cerebral infarction, Cardiac foreign body, Infective endocarditis

## Abstract

**Background:**

In this report, we describe a case of cerebral infarction caused by cardiac foreign body–induced infective endocarditis. We discuss the paradox of the treatment we used and highlight the need for careful examination of patients without histories and complaints of cardiac disease.

**Case presentation:**

Our patient was a 48-year-old Asian woman who presented with symptoms of cerebral infarction without any characteristic features of infective endocarditis. Appropriate treatment had been delayed, which made her therapy a little bit complicated. The optimal treatment of our patient was apparently surgery. However, the appropriate timing of her operation is still argued among surgeons at our department because of her acute cerebral infarction.

**Conclusions:**

Patients with cardiac foreign bodies need timely surgery, especially patients who display symptoms of nervous system or cardiovascular system imbalance. In this case report, we share our experiences with treating such a patient, which may have some clinical implications in a contradictory situation. To the best of our knowledge, this report is the first of its kind and will broaden understanding of the clinical diagnosis of this type of case.

## Background

Patients with cardiac foreign bodies usually have symptoms such as chest pain, dyspnea, fever, arrhythmia, endocarditis, and/or cardiac tamponade, or even worse symptoms [1]. Most patients are diagnosed accurately and treated promptly with the help of patients’ individual histories. However, patients who have some impairment in communication are difficult to diagnose. In this case report, we describe a patient with a history of encephalitis who had symptoms of cerebral infarction caused by foreign body–related infective endocarditis (IE). We also discuss the clinical contradictions in the therapeutic process.

## Case presentation

A 48-year-old Asian woman presented, along with her relatives, to the outpatient department of our hospital complaining of unconsciousness of 4 minutes’ duration with fecal incontinence that had occurred 20 days earlier. The patient had difficulty with communication because of a history of encephalitis. Thus, the main narrator of her history was her cousin. We were told that the patient had a history of common cold diagnosed by her local doctor 3 months earlier and had been treated with antibiotics (including ribavirin, amoxicillin sodium and clavulanate potassium, cefoperazone sulbactam, and levofloxacin) for about 1 month because of repeated fever.

**Fig. 1:**
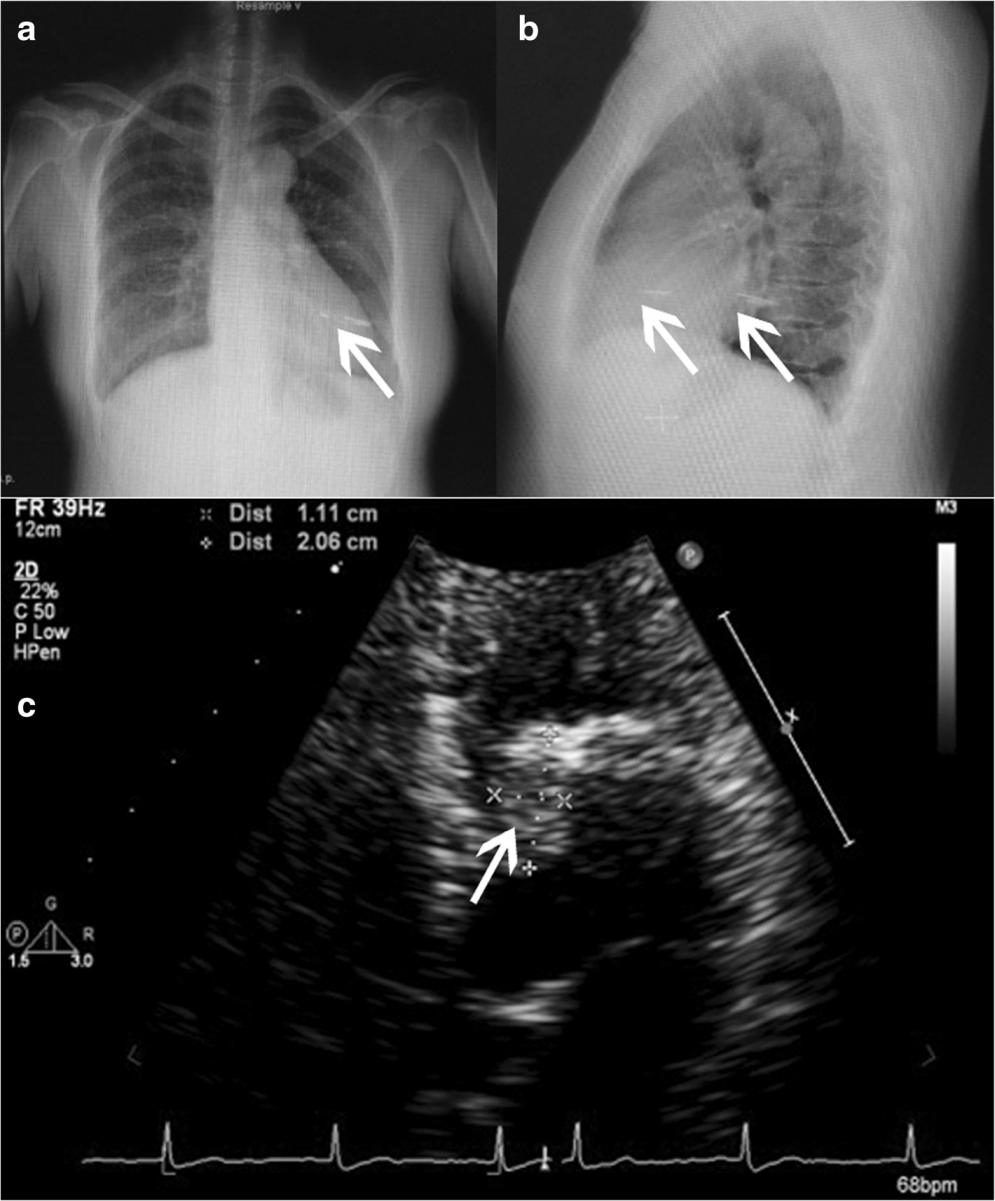
Chest x-ray and echocardiogram of the patient. **a** and **b** Chest x-rays show a needle in the heart and lung, respectively (*white arrows*). **c** Echocardiogram shows a huge echogenic mass (21 × 11 mm; *white arrow*) with some calcifications

**Fig. 2:**
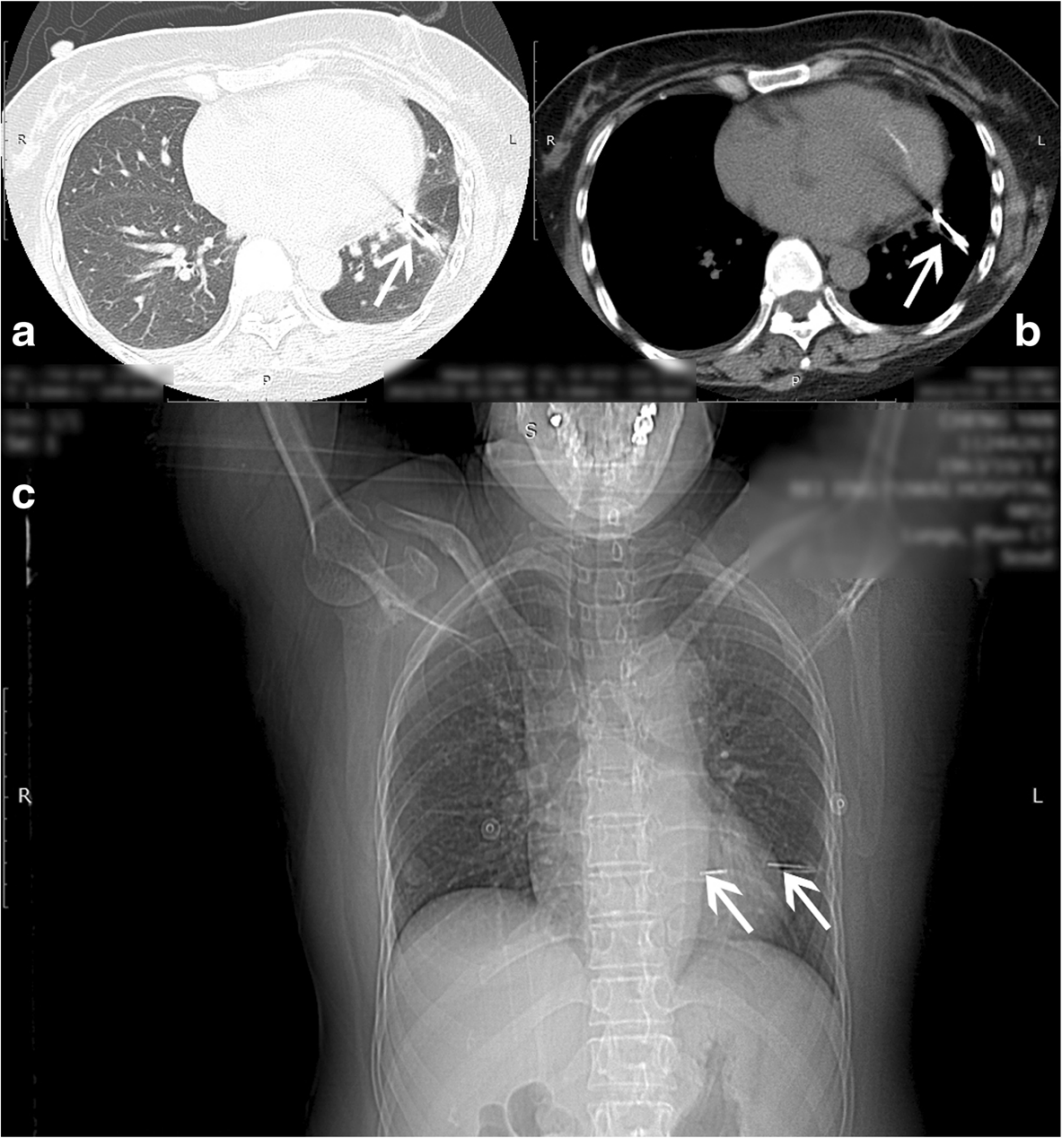
Chest computed tomographic images show the foreign body (*arrows*) inserted into the left lung and left ventricle. **a** The lung window. **b** The mediastinal window. **c** The whole chest

The patient’s temperature was normal during her hospitalization, but the results of her blood test showed elevations in the percentage of neutrophil granulocytes (78.7 %) and in her C-reactive protein level (13.2 mg/dl). Her chest x-ray showed two foreign bodies in her chest: one in the base of the left lower lobe and the other in the left ventricle (Fig. 1). Her echocardiogram showed a 21 × 11–mm echogenic mass with some calcifications attached to the anterolateral papillary muscle in the left ventricle (Fig. 1). Her chest computed tomographic (CT) scans confirmed the presence of the foreign bodies (Fig. 2). According to her cousin, she had been pierced by a foreign body through her chest wall approximately 3 months or longer before presentation.

**Fig. 3:**
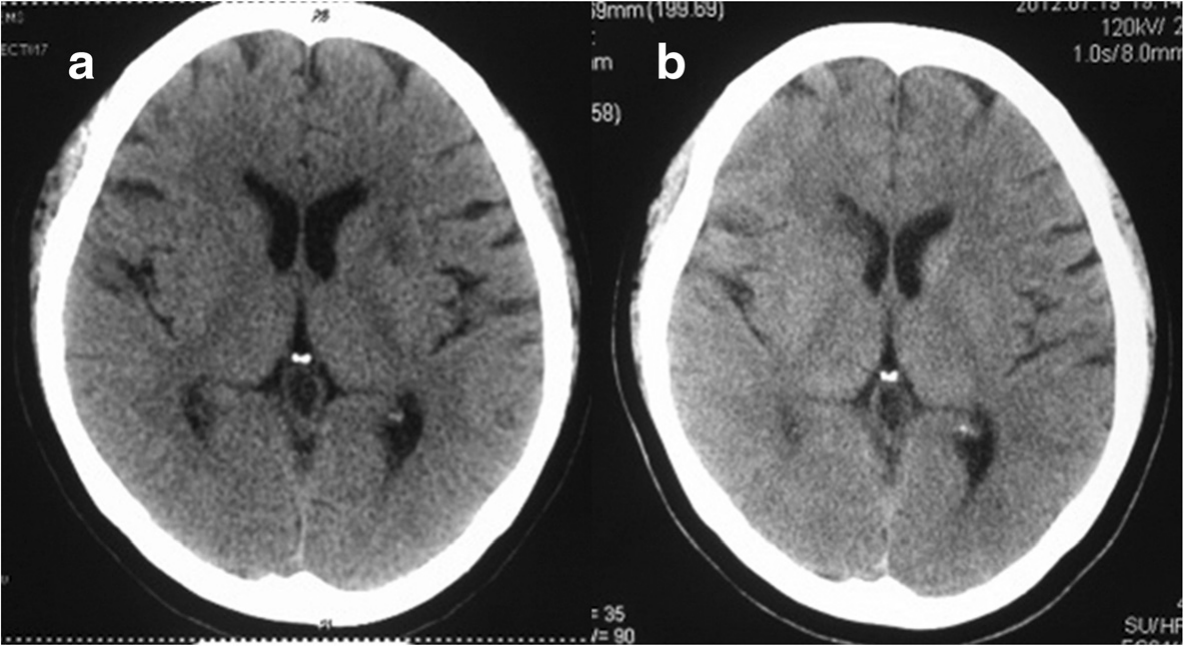
The patient’s cerebral computed tomographic scans. **a** Scan taken 9 days after the patient’s unconsciousness. **b** Scan obtained 19 days after the patient’s unconsciousness, showing improvement in her condition

**Fig. 4:**
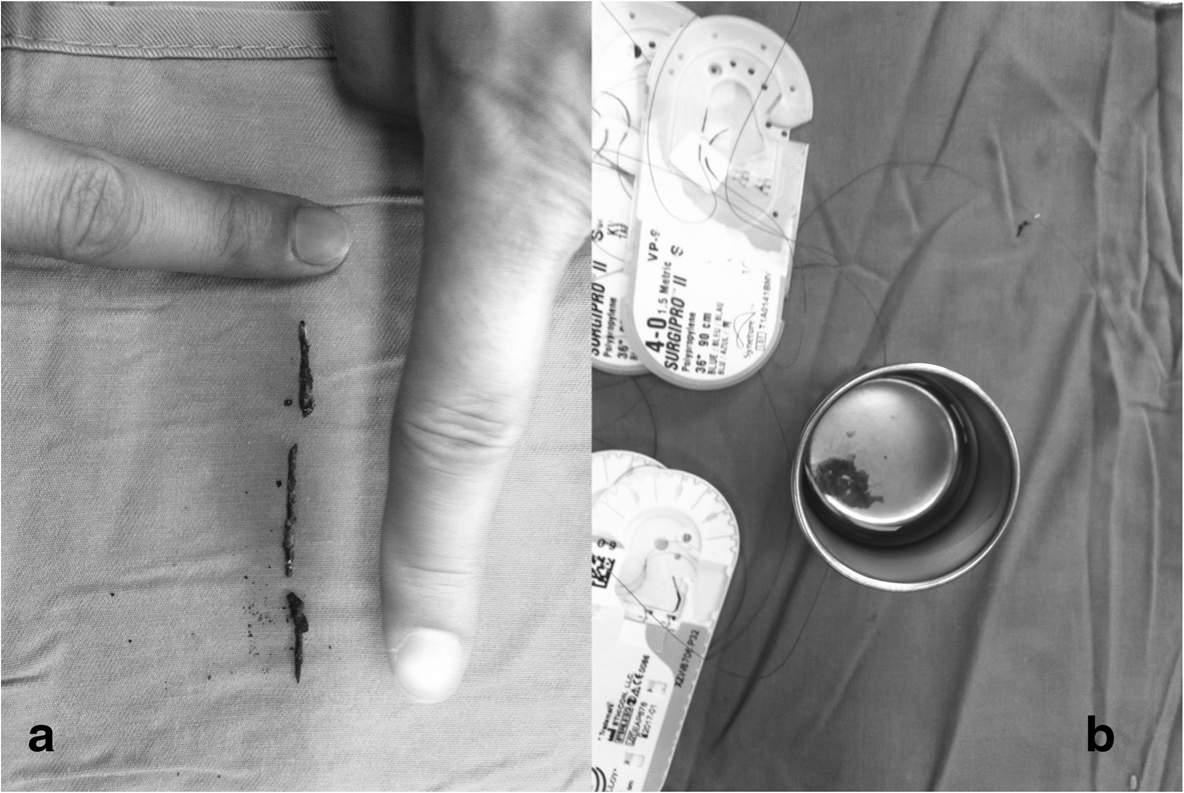
Photographs show the length of the foreign body (**a**) and the vegetation around the foreign body (**b**)

During the patient’s hospitalization, she had numbness in her left arm and drooping on the left side of her face. Serial cerebral CT scans revealed multiple new infarction areas in her brain (Fig. 3). Because of the positional relationship to the ventricle and her recurrent cerebral infarctions, surgical treatment was necessary. However, the timing of the operation was argued among surgeons in our department. A consensus was ultimately made according to the special situation of the patient. Five days after the cerebral infarction, surgery was performed to remove the foreign body from the patient’s lung and heart. During the operation, the metal foreign body was found at the bottom of the anterior papillary muscle and penetrating the left ventricular free wall to the lung with formation of a calcified sinus (Fig. 4).

One blood culture done before the patient’s surgery revealed the presence of *Staphylococcus epidermidis*, but the patient did not have any bacteremia symptoms such as chill, fever, or shock. It was disappointing that the result of the bacterial culture of the needle and myocardium was negative. Nevertheless, the antibiotic therapy was continued for 1 month after surgery on the basis of the blood culture result. The patient was discharged in good clinical condition.

## Discussion

Our patient was extremely lucky that she survived her penetrating heart injury. However, she was misdiagnosed with common cold because of fever and cough. Actually, in retrospect, these were symptoms of IE. Until she presented with neurological symptoms, IE had been diagnosed on the basis of her chest roentgenogram and echocardiogram [2, 3]. According to the European Society of Cardiology guidelines for IE, symptoms that should exclude the possibility of IE include new regurgitant heart murmur, embolic events, sepsis of unknown origin (especially if associated with an IE-causative organism), and specific fever [4].

New onset of cerebral infraction is a relative contraindication for extracorporeal circulation, which is the principal clinical contradiction in patients with cerebral infarction and IE [5]. Extracorporeal circulation may worsen a cerebral infraction, cause a nonbleeding infarction to become a bleeding infarction, aggravate hydrocephalus, and lead to pneumonia and renal failure. All of these are severe complications.

Although IE may increase the risk of embolism, treatment of foreign bodies in the heart must be individualized [6]. Actis Dato and colleagues [5] reviewed 14 cases of posttraumatic foreign bodies in the heart and found that asymptomatic foreign bodies within patients with risk factors such as embolism, infection, or arrhythmia should be removed.

Our patient underwent surgery 5 days after new-onset cerebral infarction and had difficulty in her postanesthetic recovery, which may have been related to the cerebral infarction. Fortunately, she had a satisfactory recovery and was discharged from the hospital without nervous system symptoms. Guidelines do help a lot in clinical decision-making, but individualized strategies should be used according to each patient’s clinical situation.

## Conclusions

Foreign bodies in the heart can cause lethal consequences. Therefore, surgical treatment plays an important role in the therapeutic strategy. Considering our treatment of our patient’s IE, we administered antibiotic drugs for 1 month. In retrospect, we think that both the patient’s thrombosis and her bacterial infection caused her cerebral infarction.

## Consent

Written informed consent was obtained from the patient for publication of this case report and any accompanying images. A copy of the written consent is available for review by the Editor-in-Chief of this journal.

## Abbreviations

CT: computed tomography
IE: infective endocarditis
